# Relationship between the Dynamics of Gross Composition, Free Fatty Acids and Biogenic Amines, and Microbial Shifts during the Ripening of Raw Ewe Milk-Derived Idiazabal Cheese

**DOI:** 10.3390/ani12223224

**Published:** 2022-11-21

**Authors:** Gorka Santamarina-García, Gustavo Amores, Emma López de Armentia, Igor Hernández, Mailo Virto

**Affiliations:** Lactiker Research Group, Department of Biochemistry and Molecular Biology, Faculty of Pharmacy, University of the Basque Country (UPV/EHU), Unibertsitate Ibilbidea 7, 01006 Vitoria-Gasteiz, Basque Country, Spain

**Keywords:** metagenomics, 16S rRNA gene sequencing, sheep cheese, cheese ripening, cheese quality, cheese safety, O2PLS, CCorA

## Abstract

**Simple Summary:**

The microbiota present in cheese is of special interest as it contributes to the synthesis of different compounds related to cheese quality and safety. However, to date, no studies have been carried out in cheese to elucidate the relationship between bacterial communities, characterized by high-throughput sequencing (HTS), and the dynamics of gross composition, free fatty acids (FFAs) and biogenic amines (BAs) during ripening. In this sense, this work focused on Idiazabal PDO cheese, a semi-hard or hard cheese produced from raw ewe milk. Results revealed that the non-starter lactic acid bacteria *Lactobacillus*, *Enterococcus* and *Streptococcus* were positively associated with the changes in gross composition and the release of FFAs, while only *Lactobacillus* was positively associated with the production of BAs. Several genera of environmental or undesirable bacteria presented negative correlations, which could indicate a negative impact of gross composition on their growth, the antimicrobial effect of FFAs and/or the importance of such FFAs as metabolic substrates for these bacteria, and their capability to degrade BAs.

**Abstract:**

This study reports for the first time the relationship between bacterial succession, characterized by high-throughput sequencing (sequencing of V3–V4 16S rRNA regions), and the evolution of gross composition, free fatty acids (FFAs) and biogenic amines (BAs) during cheese ripening. Specifically, Idiazabal PDO cheese, a raw ewe milk-derived semi-hard o hard cheese, was analysed. Altogether, 8 gross parameters were monitored (pH, dry matter, protein, fat, Ca, Mg, P and NaCl) and 21 FFAs and 8 BAs were detected. The ripening time influenced the concentration of most physico-chemical parameters, whereas the producer mainly affected the gross composition and FFAs. Through an O2PLS approach, the non-starter lactic acid bacteria *Lactobacillus*, *Enterococcus* and *Streptococcus* were reported as positively related to the evolution of gross composition and FFAs release, while only *Lactobacillus* was positively related to BAs production. Several environmental or non-desirable bacteria showed negative correlations, which could indicate the negative impact of gross composition on their growth, the antimicrobial effect of FFAs and/or the metabolic use of FFAs by these genera, and their ability to degrade BAs. Nonetheless, *Obesumbacterium* and *Chromohalobacter* were positively associated with the synthesis of FFAs and BAs, respectively. This research work provides novel information that may contribute to the understanding of possible functional relationships between bacterial communities and the evolution of several cheese quality and safety parameters.

## 1. Introduction

Idiazabal cheese is a semi-hard or hard cheese from the Basque Country (Southwestern Europe), which is produced from the raw milk of Latxa and/or Carranzana sheep and with at least 60 days of ripening. Its production is regulated by its Protected Designation of Origin (PDO) [1]. Like other raw milk cheeses, the aromatic profile of this cheese is richer compared to those produced from pasteurized milk [2,3,4,5], which has been mainly attributed to the indigenous milk microbiota [6,7,8,9]. Nowadays, high-throughput sequencing (HTS) is a powerful technology to characterize the bacterial communities of fermented foods, avoiding the limitations of culture-based methods [10]. However, these technologies also have their limitations, such as the accuracy in taxonomic identification at the species level or the high associated cost due to equipment, reagents or trained personnel [11]. In a previous study, the microbiota of Latxa ewe raw milk and the bacterial shifts that occur during the production and ripening of Idiazabal cheese have been successfully described by means of this technology. In fact, some bacterial genera not reported so far in raw ewe milk and cheese have been identified. It should be noted that notable differences were observed among producers, which was attributed to the different practices carried out among producers in relation to herd management, such as feeding; or cheese making, for example, the type of rennet used or the technological settings [12].

The microbiota of cheese is of great importance since it contributes to numerous biochemical reactions involved in the formation of compounds related to cheese quality and safety [13,14,15]. Lipolysis is one of the most important processes for flavour development during cheese ripening. It is defined as the enzymatic hydrolysis of milk triglycerides (TG) with the subsequent release and accumulation of free fatty acids (FFAs). FFAs contribute directly to cheese flavour, but also act as precursor molecules for subsequent reactions that lead to the production of volatile compounds [16,17]. The most important lipolytic agents in cheese are lipoprotein lipase (LPL) from raw milk, rennets containing pregastric lipase and microbial lipases and esterases [17,18,19].

Proteolysis is another important biochemical event that takes place during ripening, whereby caseins are broken down into peptides and free amino acids (FAAs). Therefore, and together with the metabolism of the resulting peptides and FAAs, it contributes to cheese texture and flavour [18,20]. However, microbial decarboxylation of FAAs could lead to the production of biogenic amines (BAs), which are non-volatile low molecular weight nitrogenous organic bases with biological activity [21,22,23]. BAs are not harmful at low levels, but some can have toxicological effects after ingestion in high concentrations [24,25]. In relation to dairy products and specifically cheese, histamine and tyramine are the main ones responsible for intoxication. Histamine causes the so-called “histamine poisoning”, characterized by low blood pressure, skin irritation or rashes typical of allergic reactions; while tyramine causes the so-called “cheese reaction”, with symptoms like migraines, headaches or blood pressure increase. Putrescine and cadaverine are also important BAs, since they can potentiate the toxic effect of other BAs due to the inhibition of detoxifying amine oxidases and are related to the production of carcinogenic nitrosamines [24,26,27]. Within fermented foods, cheese is classified as a potential source of BAs due to the high microbial activity associated [23,28]. Nevertheless, so far, no legal limits have been established [29,30].

To elucidate the relationship between bacterial communities and the chemical composition of fermented foods, complex chemometric approaches are needed [31,32,33]. The multivariate bidirectional orthogonal partial least squares (O2PLS) [34] is one of the most useful approaches [35,36,37]. It has been successfully applied to analyse the correlation between bacterial and fungal metabolisms and different metabolites, such as FAAs, of fermented foods [38,39,40,41]; including cheese [31,32]. However, its combination with other parameters, such as correlation coefficients, is considered the most appropriate approach to studying such relationships [42,43]. Recently published studies analyse the relationship between bacterial communities and the chemical composition of different types of cheeses [14,31,44,45]. However, information on raw ewe milk-derived cheeses is scarce [46,47,48]. Moreover, to the best of our knowledge, no study has analysed the relation between bacterial succession characterized by HTS and the evolution of gross composition, FFAs and BAs of any type of cheese. To date, no multivariate approach and only some correlations have been reported between specific bacteria, such as *Lactobacillus*, and certain physico-chemical parameters, such as pH, NaCl, short-chain FFAs, such as C4; or histamine [49,50,51].

Therefore, the objective of the present study was to analyse how the gross composition, FFAs and BAs content of Idiazabal cheese evolve during the ripening time and to explore how they relate with the bacterial communities present. Moreover, the potential differences among producers producing the same type of cheese were also analysed.

## 2. Materials and Methods

### 2.1. Cheese Sampling

The Idiazabal PDO cheeses analysed in the present study were produced by four artisanal producers (identified as A, B, C and D), whose dairies were situated in different geographic locations throughout the Basque Country. Idiazabal cheeses were produced from the raw milk of Latxa sheep, each producer employing the milk of its own flock and following the specifications issued by the Idiazabal Designation of Origin Regulatory Board [52]. The producers employed the mesophilic lyophilized starter culture Choozit MM 100 LYO 50 DCU (mixture of *Lactococcus lactis* subsp. *lactis*, *Lactococcus lactis* subsp. *cremoris* and *Lactococcus lactis* subsp. *lactis* biovar. *diacetylactis*) (DuPont NHIB Ibérica S.L., Barcelona, Spain). The milk was coagulated either with artisanal lamb rennet (produced as described previously [53]) or with commercial NATUREN^®^ 195 Premium rennet (Chr. Hansen Holding A/S, Hørsholm, Denmark). Cheese ripening took place in chambers with 80–95% relative humidity and 8–14 °C temperature. The cheeses were collected in duplicate at six ripening times (1, 7, 14, 30, 60 and 120 days). Therefore, a total of 48 cheese samples were analysed. The samples were collected and transported in refrigeration (3 °C) to the laboratory. Each cheese was divided in eighths and the analyses of gross composition, except NaCl, were performed on fresh samples, while for the subsequent determination of NaCl and the analysis of FFAs and BAs the samples were stored in a freezer (−80 °C). The samples were allowed to thaw at 5 °C for 24 h and then, kept at room temperature for 1 h prior to analysis.

### 2.2. Analysis of Gross Composition

The pH was measured in a pH-meter micropH 2000 (Crison Instruments S. A., Barcelona, Spain). Dry matter, fat, protein, calcium, magnesium and phosphorous content was measured using the SpectraAlyzer 2.0 FOOD Near Infrared Spectrometer (NIR) (ZEUTEC GmbH, Rendsburg, Germany), as described by Aldalur et al. [54]. NaCl content was determined on the basis of the standard method ISO 5943 IDF 88 [55], but replacing the potentiometric determination of the endpoint with its colorimetric determination (Mohr titration method). These analyses were conducted in duplicate.

### 2.3. Analysis of Free Fatty Acids

FFAs were extracted, isolated, identified and quantified without derivatization by gas–liquid chromatography (GLC), basically as described by Chavarri et al. [56]. In short, 0.5 g of cheese sample was ground with 3.0 g of anhydrous Na_2_SO_4_ (reagent grade, Scharlab, Barcelona, Spain), and 0.3 mL of 2.5 M H_2_SO_4_ (reagent grade, Scharlab, Barcelona, Spain) and 100 μL of internal standard (IS) solution (n-pentanoic (5:0), n-nonanoic (9:0) and n-heptadecanoic (17:0) acids (GC grade, Sigma-Aldrich, Madrid, Spain), 1 mg/mL solution of each FFA in diethyl ether-heptane 1:1, *v*/*v* (GC grade, ROMIL Ltd., Cambridge, UK)) were added. The lipids were extracted three times with 3.0 mL of diethyl ether-heptane 1:1, *v*/*v* each time. After each extraction, the solution was cleared by centrifugation. The three extracts were pooled and applied to an aminopropyl-bonded phase column (Sep-Pak^®^, 3 cm^3^, 500 mg, Waters, Barcelona, Spain), previously equilibrated with 10.0 mL of heptane. The TG were eluted with 10.0 mL of chloroform-isopropanol 2:1, *v*/*v* (GC grade, ROMIL Ltd., Cambridge, UK) and the FFAs were isolated by elution of 5.0 mL of diethyl ether-formic acid [reagent grade, Panreac Química, Barcelona, Spain (98:2, *v*/*v*)].

FFAs were separated on an FFAP capillary column (25 m length, 0.32 mm inner diameter, 0.50 μm film thickness) (Agilent Technologies, Madrid, Spain) installed on a 7890 Series II gas chromatograph (Agilent Technologies, Madrid, Spain) equipped with a flame ionization detector. Helium (99.999% purity, Air liquid, Madrid, Spain) was used as carrier gas at a flow rate of 2 mL/min. The temperature was raised from 65 °C to 240 °C at 10 °C/min, and this final temperature was maintained for 30 min. The split/splitless ratio was set at 1:5. The injector and detector temperatures were maintained at 325 °C and 275 °C, respectively. FFAs were identified by comparison of their retention times with those of high purity standards (≥90%; Sigma-Aldrich, Madrid, Spain). Quantification was performed by the internal calibration method for each of the FFAs found in the samples. Relative IS response factors were calculated for the standard solutions of FFAs. The concentrations of FFAs in the cheese samples were expressed as µmol/g of cheese.

### 2.4. Analysis of Biogenic Amines

BAs were extracted, separated, identified and quantified by reversed phase-high-performance liquid chromatography (RP-HPLC), based on the method described by Busto et al. [57]. In brief, 1.0 g of cheese was grounded together with 9.0 mL of trichloroacetic acid (TCA) 5%, *w*/*v* (reagent grade, Scharlab, Barcelona, Spain), after which, 1.0 mL of TCA 5% was added, which contained 1,7-diaminoheptane 1.5 mM (≥98%, Sigma-Aldrich, Madrid, Spain) employed as IS. The acid extract was sonicated for 25 min and centrifuged at 5000 rpm for 30 min at room temperature. The extract was filtered through Durapore filters (0.45 mm pore size; Millipore, Madrid, Spain). The derivate reagent was formed by reconstituting 6-aminoquinolyl-N-hydroxy-succinimidyl carbamate (reagent grade, Waters, Barcelona, Spain) with 1.0 mL of acetonitrile (reagent grade, Waters, Barcelona, Spain) according to the specifications of the AccQ-Fluor Reagent Kit (Waters, Barcelona, Spain). Then, 2.5 µL of the amine extract was mixed with 75 µL of borate buffer (pH 8.8, reagent grade, Waters, Barcelona, Spain) and 25 µL of the derivative reagent AQC (reagent grade, Waters, Barcelona, Spain). The mixture was shacked and kept at room temperature for 1 min and finally, maintained at 55 °C for 10 min.

The derivatized BAs were separated in an XTerraTM MS C18 column (XTerra MS C18 Column, 125 Å pore size, 5 µm particle size, 4.6 mm i.d. × 250 mm length) installed in an Alliance 2690 separation module, equipped with a quaternary pump system, automatic injector, vacuum degasification, thermostated oven and with a Waters 474 Scanning Fluorescence Detector (Waters, Barcelona, Spain). For separating BAs, both the column and the pre-column (XTerra MS C18 Sentry Guard Cartridge, 125 Å, 5 µm, 3.9 mm × 20 mm) were maintained at 50 °C and two solvents were used: (A) 0.05 M sodium acetate solution in 1% tetrahydrofuran pH 7.0 (>99.9%, ROMIL Ltd., Cambridge, UK) and (B) methanol (>99.9%, ROMIL Ltd., Cambridge, UK). The programme is shown in Supplementary Table S1. Subsequently, the identification was made by fluorimetry, at excitation and emission wavelength of 250 nm and 395 nm, respectively. The quantification was performed using 1,7-diaminoheptane (≥98%, Sigma-Aldrich, Madrid, Spain) as IS and analysing standard solutions with known concentrations of methylamine (MMA), α-aminobutyric acid (AABA), γ-aminobutyric acid (GABA), cadaverine (CAD), ethanolamine (MEA), ethylamine (ETY), histamine (HIS), methylbutylamine + spermidine (MBA + SPD) (those were analysed together because it was not possible to separate peaks), n-butylamine (NBA), phenylethylamine (PHE), putrescine (PUT), spermine (SPM) and tyramine (TYR) (reagent grade, Sigma-Aldrich, Madrid, Spain). The concentrations of BAs in cheese samples were expressed as µmol/g of cheese.

### 2.5. Statistical Analysis

Different packages and software were employed for physico-chemical parameters (gross composition, FFAs and BAs) analysis and three significant figures were used to express the results. The IBM SPSS statistical package version 26.0 (IBM SPSS Inc., Chicago, IL, USA) was used for data preparation and analysis. Kruskal–Wallis analysis of variance with Bonferroni correction was performed using the IBM SPSS package to determine the effect of ripening time and producer on each physico-chemical parameter. Permutational multivariate analysis of variance (PERMANOVA) was performed using RStudio version 1.3.959 and R version 3.6.3 [58] through the “vegan” package [59]. The objective was to measure the overall effect of the aforementioned factors on the physico-chemical parameters. The F statistic was employed to measure the influence of each factor. Physico-chemical parameters were selected, log-transformed, when necessary, and unit variance (UV) scaled, and a heat-map with hierarchical clustering analysis (HCA) was performed using the “gplots” package of R [60]. The aim was to analyse the clustering of physico-chemical parameters during ripening time. Trends along ripening were then explored in depth by means of a principal component analysis (PCA) performed using the SIMCA software version 15.0.0.4783 (Umetrics AB, Umeå, Sweden). The number of principal components (PCs) to be taken into account was decided on the basis of eigenvalues (greater than 1.0) and cross-validation. An orthogonal partial least squares discriminant analysis (OPLS-DA) was run in SIMCA software in order to analyse whether samples differ among producers. Variable influence on projection (VIP) and loadings weights were employed to estimate the importance of each physico-chemical parameter in the model.

### 2.6. Correlation with Bacterial Communities

To characterize the bacterial communities and the shifts that occur during the ripening of the collected Idiazabal cheese samples, an HTS analysis was performed, as previously described [12]. Briefly, 10 g of cheese was added to 90 mL of 2% (*w*/*v*) sterile sodium citrate (pH 8.0), and homogenized in a sample homogenizer (Masticator Basic 400; IUL Instruments, Königswinter, Germany) six times (cycles of 20 s ON and 10 s OFF each). Of the resulting dispersion, 1.5 mL was centrifuged (8000 *g* for 10 min at 4 °C) and the supernatant containing the fat was discarded. The pellet obtained was resuspended in 600 µL of sodium citrate, and centrifuged thrice (8000 *g* for 10 min at 4 °C). From the resulting product, DNA was extracted by means of the DNeasy Blood & Tissue Kit (Qiagen, Valencia, CA, USA) and the 16S rRNA gene library was prepared with the Nextera XT DNA Library Preparation Kit (Illumina Inc., San Diego, CA, USA). The hypervariable V3–V4 regions of the 16S rRNA gene were amplified by means of PCR (forward primer: 5′-TCGTCGGCAGCGTCAGATGTGTATAAGAGACAGCCTACGGGNGGCWGCAG-3′; reverse primer: 5′-GTCTCGTGGGCTCGGAGATGTGTATAAGAGACAGGACTACHVGGGTATCTAATCC-3′), as described by Klindworth et al. [61]. Next, the gene encoding 16S rRNA was sequenced with the MiSeq Reagent Kit v3 (2 × 300 bp) (Illumina Inc.) on the Illumina MiSeq platform. Quality filtering and trimming of raw reads was performed with MiSeq Reporter software, and the MG-RAST web data analysis tool [62] was used for taxonomic classification, based on the Silva SSU database [63]. Bacterial abundance was given as relative abundance (%) based on the identified sequences.

To study the relationship between the identified bacterial genera and the physico-chemical parameters, an O2PLS approach was applied to log-transformed, when necessary, and UV scaled data in SIMCA, selecting the main bacterial genera as X-variables and physico-chemical parameters as Y-variables. The model was validated, among others, by R2 and Q2 values, permutation test or inner relation plot. Correlations were also analysed by Spearman’s rank correlation coefficients calculated using SPSS and displayed as a heat map with an HCA in R, as previously mentioned. The canonical correlation analysis (CCorA) multivariate statistical approach was performed using the “vegan” package of R to verify the previously obtained correlations. 

## 3. Results

### 3.1. Changes in Gross Composition

**Figure 1:**
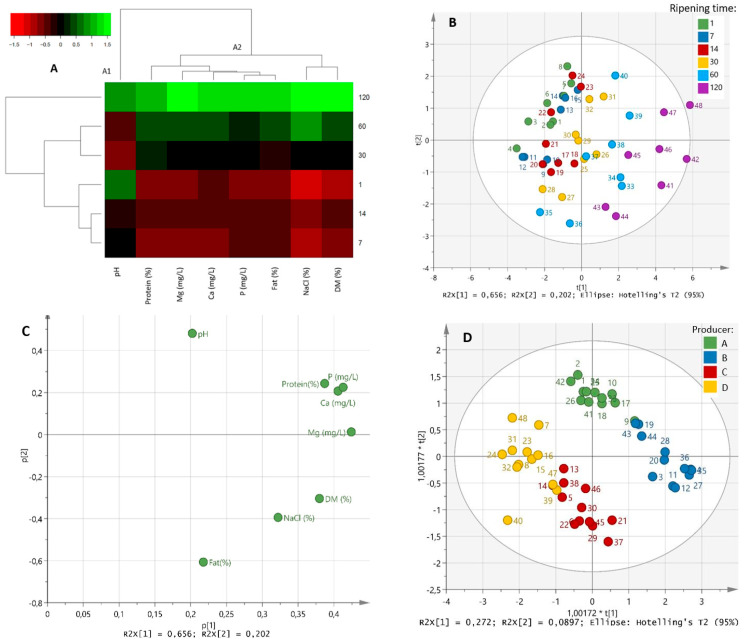
HCA heat map (**A**), scores and loadings plots of PCA (**B** and **C**, respectively) and scores plot of the OPLS-DA based on the producer of the analysed gross composition parameters during ripening (1, 7, 14, 30, 60 and 120 days) (**D**). The scale values of the HCA correspond to log-transformed and UV scaled data.

Firstly, the evolution of gross composition during the ripening of Idiazabal cheeses was analysed. As shown in Figure 1A and Supplementary Table S2, the pH showed a different evolution from the rest of the parameters, that is why it formed a single cluster (A1). The pH was 5.08 ± 0.08 on the first day of ripening, which decreased and reached the lowest values at 30 days (4.91 ± 0.14). Then, up to 120 days, the pH increased close to the initial values (5.11 ± 0.14) (*p* ≤ 0.05). The rest of the parameters presented an increasing trend throughout the ripening time (cluster A2), namely dry matter, protein, fat, NaCl, Ca, Mg and P content (*p* ≤ 0.01). It should be noted that changes differed largely according to the producer (*p* ≤ 0.05), except for the NaCl content (*p >* 0.05).

By means of a multivariate approach, PERMANOVA indicated that both the ripening time and the producer factors influenced the gross composition of Idiazabal cheeses (*p* ≤ 0.001) (data not shown). However, the F statistic indicated that the ripening time had a greater impact than that of the producer (38.1 and 7.89, respectively). Through a PCA, which revealed three PCs and accounted for 94.8% of the variance, these results were confirmed. According to the scores plot (Figure 1B), PC1 values ranged from negative (for less ripened cheeses) to positive (for more ripened cheeses). Consequently, PC1 (accounting for 65.6% of the explained variance) was clearly correlated with the ripening time. Gross composition parameters showed positive loadings in PC1, which confirmed that their evolution was clearly affected by the ripening time (Figure 1C). On the other hand, PC2 (accounting for 20.2% of the explained variance) was related to the producer factor, indicating that the gross composition also differed according to the dairy, although to a lesser extent (Figure 1B). Differences among producers were confirmed through an OPLS-DA, which reported a clear distinction among all producers (Figure 1D). The pH and protein content were reported as important parameters for such differentiation.

### 3.2. Changes in Free Fatty Acids

**Figure 2:**
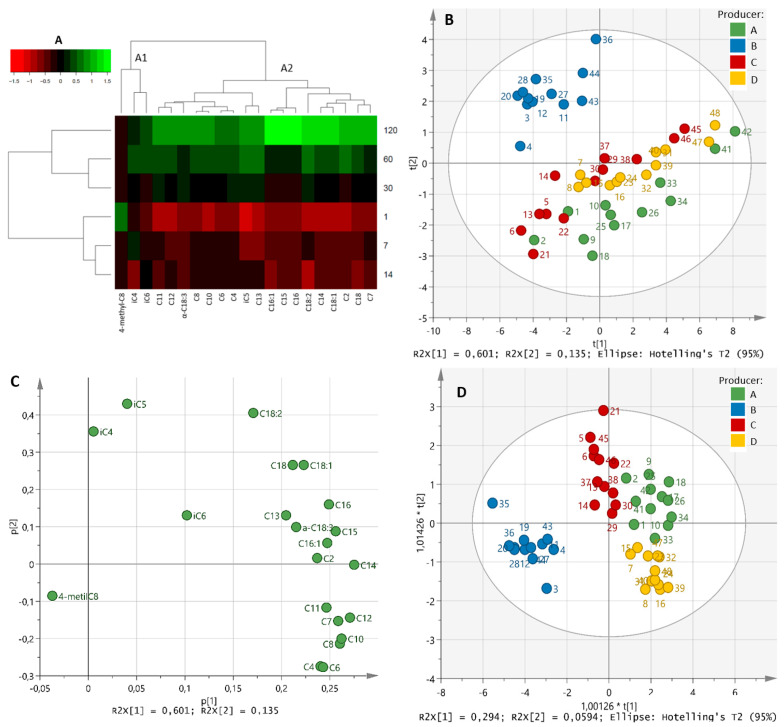
HCA heat map (**A**), scores and loadings plots of PCA (**B** and **C**, respectively) and scores plot of the OPLS-DA based on the producer of the identified FFAs during ripening (1, 7, 14, 30, 60 and 120 days) (**D**). The scale values of the HCA correspond to log-transformed and UV scaled data.

As shown in Table 1, the concentrations of total, unsaturated, saturated, short-, medium- and long-chain FFAs of Idiazabal cheeses increased throughout the ripening time (*p* ≤ 0.01). Total FFAs varied from 12.0 ± 5.95 µmol/g in 1-day-old ripened cheeses to 49.9 ± 24.5 µmol/g in 120-day-old ripened cheeses (Table 1, Supplementary Figure S1). Along the whole process, saturated and short-chain FFAs predominated. Individually, a total of 21 FFAs were identified and C2, C4, C6 and C10 were the most abundant (Supplementary Table S3). A clear increase in the concentrations of all individual FFAs was observed during ripening time (*p* ≤ 0.05), except for iC4, iC6 and 4-methyl-C8 (*p >* 0.05). That is why the HCA analysis reported two different clusters (Figure 2A). Clear differences were observed among producers, which, in general, increased as ripening progressed (Table 1, Supplementary Table S3).

PERMANOVA confirmed that ripening time and producer factors influenced the lipolysis of Idiazabal cheeses (*p* ≤ 0.001) (data not shown), although the ripening effect was greater (14.3 and 11.8, respectively). PCA revealed two PCs, accounting for 73.7% of the variance. PC1 values ranged from negative (for less ripened cheeses) to positive (for more ripened cheeses) (Figure 2B). Therefore, PC1 (accounting for 60.1% of the explained variance) was correlated to the ripening time. All FFAs, except 4-methyl-C8, iC4, iC5 and iC6, showed positive loadings in PC1, confirming an increase in their concentrations during the ripening time (Figure 2C). PC2 (accounting for 13.5% of the explained variance) was related to the producer, indicating a notable but lower impact than that of the ripening time. Differences among producers were confirmed by an OPLS-DA, which reported a clear differentiation between producer B, who used commercial rennet, and the rest that employed artisanal rennet (Figure 2D). C4, C6, C8, C10 and C12 were reported as important FFAs for such differentiation. It is noteworthy that, although to a lesser extent, a distinction among the producers who used artisanal rennet (A, C and D) was also observed (Figure 2D).

### 3.3. Changes in Biogenic Amines

**Figure 3:**
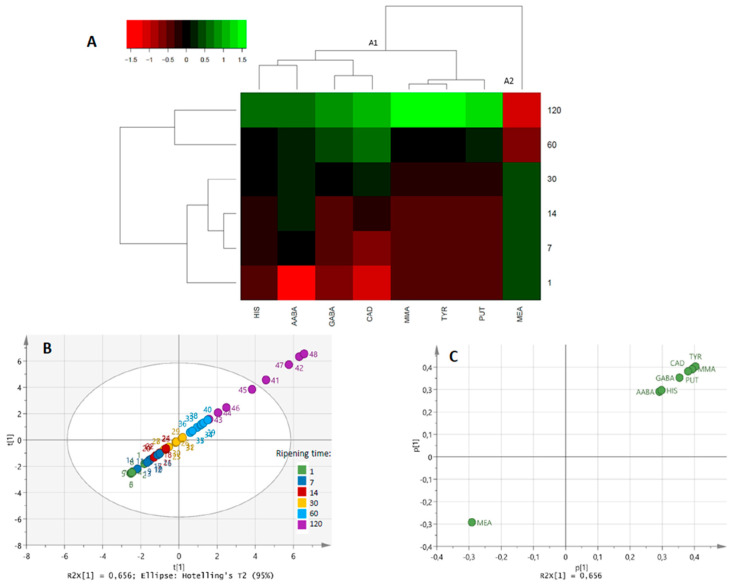
HCA (**A**) and scores and loadings plots of PCA (**B** and **C**, respectively) of the identified BAs during ripening (1, 7, 14, 30, 60 and 120 days) of Idiazabal cheese. The scale values of the HCA correspond to log-transformed and UV scaled data.

Of the 14 BAs analysed, only AABA, GABA, CAD, HIS, MEA, MMA, PUT and TYR were detected during the ripening of Idiazabal cheese (Figure 3 and Supplementary Table S4). Total BAs concentration varied from a mean of 4.29 ± 0.375 µmol/g at 1 day of ripening, to 14.9 ± 3.73 µmol/g at 120 days. As shown in Figure 3A, the concentration of most individual BAs increased during ripening (cluster A1). However, MEA presented a different trend, remaining constant at 0.836 ± 0.0573 µmol/g until 30 days of ripening and, in general, decreasing from that moment on (cluster A2). Individually, MMA was the most abundant (3.16 ± 0.323 µmol/g at 120 days of ripening), followed by PUT (2.72 ± 1.39 µmol/g), CAD (2.58 ± 0.380 µmol/g) and GABA (2.35 ± 1.12 µmol/g). The ripening time effect was significant for all BAs (*p* ≤ 0.01), except for HIS (*p >* 0.05). Differences among producers were only observed for HIS (*p* ≤ 0.001).

Overall, PERMANOVA confirmed that the ripening time had a significant effect on the BAs concentrations of the analysed Idiazabal cheeses (*p* ≤ 0.001). The effect of the producer was not significant (*p >* 0.05) (data not shown). PCA revealed a unique PC accounting for 65.6% of the variance, which was highly correlated with the ripening time (Figure 3B). Consequently, samples were distributed from the third quadrant to the first one as the ripening progressed. All BAs, except MEA, were distributed together with the more ripened cheeses (Figure 3C), confirming the aforementioned dynamics. Nonetheless, a differentiation between the samples could be observed at 120 days of ripening (Figure 3B).

### 3.4. Correlation between Bacterial Dynamics and Physico-Chemical Parameters Evolution

From our previous publication, in general, it was observed that the starter LAB (SLAB) *Lactococcus* was predominant during ripening. However, after 30 or 60 days of ripening, its abundance decreased and the non-starter LAB (NSLAB) began to proliferate. Specifically, the proliferation of *Lactobacillus* was promoted in all producers (from a mean of 0.0949% at 1 day of ripening to 8.96% at 120 days), while for the rest it depended on the producer. That is, *Leuconostoc* proliferation was promoted in producer A (from 4.48% to 31.0%), while in producers A and D, *Streptococcus* and *Enterococcus* growth was promoted (from 0.750% to 4.52% and from 0.675% to 2.12%, respectively). On the other hand, the abundance of those bacteria classified as environmental or non-desirable decreased during ripening, except for some genera that remained abundant (>1%), namely *Obesumbacterium*, *Hafnia*, *Staphylococcus*, *Buttiauxella*, *Psychrobacter*, *Raoultella*, *Serratia*, *Brevibacterium* and *Erwinia*. Among these genera, the abundance of *Buttiauxella* decreased during ripening, while that of *Staphylococcus* increased and the rest showed an increase at intermediate points (at 7, 14 or 30 days of ripening). More details can be found in Santamarina-García et al. [12].

An O2PLS approach with Spearman’s rank correlation coefficients was applied to analyse the relationship between the identified main bacterial genera and the evolution of gross composition, FFAs and BAs during ripening. The key functional microbiota was identified based on: (i) the VIP value of main bacterial genera higher than 1; (ii) the loading weights of main bacterial genera and (iii) the significant (*p* ≤ 0.05) Spearman’s rank correlation coefficients, which were interpreted as follows: |ρ| < 0.500, low correlation; 0.500 ≤ |ρ| < 0.600, moderate correlation; 0.600 ≤ |ρ| < 0.700, high correlation; and |ρ| ≥ 0.700, strong correlation. It is noteworthy that many factors influence the physico-chemical parameters of Idiazabal cheese, such as the raw milk or the rennet used. Hence, the coefficients obtained are not as high as those reported for other fermented foods where microbial metabolisms are solely responsible [31,64].

#### 3.4.1. Correlation between Main Bacterial Genera and Gross Composition

**Figure 4:**
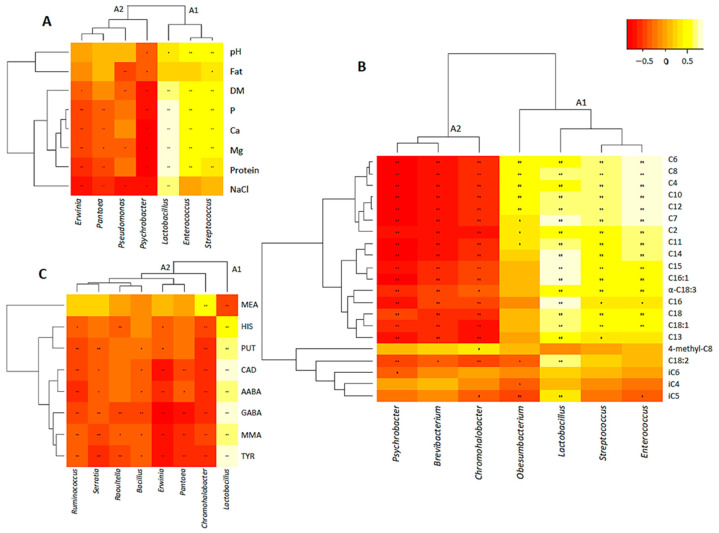
Spearman’s rank correlations with HCA analysis between key bacterial genera and gross composition parameters (**A**), FFAs (**B**) and BAs (**C**). Significant correlations are represented by ** *p* ≤ 0.01 and * *p* ≤ 0.05.

The key bacterial genera related to gross composition evolution were *Lactobacillus*, *Psychrobacter*, *Erwinia*, *Enterococcus*, *Pseudomonas*, *Pantoea* and *Streptococcus* (Figure 4A). The HCA analysis divided these bacteria into two clusters: the A1, which includes lactic acid bacteria (LAB) that were positively correlated to gross composition parameters; and A2, which includes the environmental or non-desirable genera that were negatively correlated. Within LAB, *Lactobacillus* presented the greatest positive correlations, especially with protein (ρ = 0.842), Ca (ρ = 0.809), P (ρ = 0.805) and Mg content (ρ = 0.781) (*p* ≤ 0.01). Moreover, *Lactobacillus* was the unique LAB that presented a strong positive correlation with NaCl (ρ = 0.709, *p* ≤ 0.01), whereas *Streptococcus* and *Enterococcus* were correlated to the pH (ρ ≥ 0.528, *p* ≤ 0.01). On the other hand, environmental and non-desirable bacteria showed diverse negative correlations, but all were negatively correlated with NaCl (ρ ≤ −0.531, *p* ≤ 0.01). In general, *Psychrobacter* was the most negatively correlated bacteria, with strong negative correlations with protein (ρ = –0.762), Ca (ρ = –0.750), Mg (ρ = –0.747), P (ρ = –0.718) and dry matter content (ρ = –0.705) (*p* ≤ 0.01). Overall, pH and fat content were the parameters least related to bacterial genera. Through a CCorA analysis, the aforementioned correlations were confirmed (Supplementary Figure S2).

#### 3.4.2. Correlation between Main Bacterial Genera and FFAs

Seven bacterial genera were reported as the key microbiota related to FFAs evolution, namely, *Psychrobacter*, *Brevibacterium*, *Lactobacillus*, *Enterococcus*, *Chromohalobacter*, *Streptococcus* and *Obesumbacterium* (Figure 4B). As for gross composition, the HCA analysis reported two clusters: A1 grouped those LAB and the environmental *Obesumbacterium* that were positively correlated to most FFAs concentrations and A2 those non-desirable and environmental bacteria that were negatively correlated. Within LAB, *Lactobacillus* and *Enterococcus* showed the highest correlations with FFAs concentrations (Figure 4B). *Enterococcus* showed strong correlations with short-chain FFAs, such as C4 (ρ = 0.819), C6 (ρ = 0.800), C8 (ρ = 0.780) and C10 (ρ = 0.762) (*p* ≤ 0.01). *Lactobacillus* presented the strongest correlations with C7 (ρ = 0.718) and medium- and long-chain FFAs, such as C14 (ρ = 0.745), C15 (ρ = 0.729) and C16 (ρ = 0.762) (*p* ≤ 0.01). Within environmental and non-desirable bacteria, *Obesumbacterium* showed positive correlations with short-chain FFAs, such as C4 (ρ = 0.534) and C6 (ρ = 0.502) (*p* ≤ 0.01), while the rest presented several negative correlations (*p* ≤ 0.01). As for gross composition, *Psychrobacter* was reported as the most negatively related. It showed strong negative correlations with C4 (ρ = –0.759), C6 (ρ = –0.765), C8 (ρ = –0.757) and C11 (ρ = –0.762) (*p* ≤ 0.01), for example. In addition, iC4, iC5, iC6 and 4-methyl-C8 were the FFAs least related to bacterial genera. These correlations were confirmed by means of a CCorA model (Supplementary Figure S3).

#### 3.4.3. Correlation between Main Bacterial Genera and BAs

Eight bacterial genera were reported as key bacteria related to BAs evolution, namely, *Lactobacillus*, *Erwinia*, *Chromohalobacter*, *Pantoea*, *Bacillus*, *Ruminococcus*, *Serratia* and *Raoultella* (Figure 4C). *Lactobacillus* was the only genus that presented several positive correlations, so it formed a single cluster (A1) differentiated from the rest that were mainly negatively related (A2). Individually, *Lactobacillus* was strongly correlated with the concentrations of TYR (ρ = 0.865), GABA (ρ = 0.840) and CAD (ρ = 0.752), and to a lesser extent, AABA (ρ = 0.729) and MMA (ρ = 0.704) (*p* ≤ 0.01) (Figure 4C). Within environmental and non-desirable bacteria, *Chromohalobacter* presented a high positive correlation with MEA (ρ = 0.625, *p* ≤ 0.01) and the rest showed only several negative correlations. *Erwinia* was reported as the most negatively related, mainly with GABA (ρ = –0.768), MMA (ρ = –0.651), CAD (ρ = –0.643) and TYR (ρ = –0.635) (*p* ≤ 0.01). In general, MEA was the BA correlated with fewer bacterial genera. The CCorA model confirmed the observed correlations (Supplementary Figure S4).

## 4. Discussion

It is well known that the microbiota inhabiting cheese affects the quality and safety of the final product [65]. In recent years, several studies have been published focused on elucidating the relationship between the microbiota and the production of metabolites, such as volatile compounds or FAAs, in fermented foods [38,39,66], including cheese [32,67]. However, only a few studies have analysed raw ewe milk-derived cheeses [46,47,48]. The present study aimed to elucidate the relationship between bacterial communities characterized by HTS and the evolution of gross composition, FFAs and BAs during the ripening of raw ewe milk-derived Idiazabal cheese. To the best of our knowledge, this approach has not been applied so far for any type of cheese.

Within the gross composition, the decrease in the pH until 30 days of ripening and the subsequent increase, together with the concentration of the rest of the parameters during ripening, is in agreement with what has been previously reported for Idiazabal cheese [19,50,68,69]. However, it is noteworthy that the evolution of Ca, Mg and P content during ripening has not been studied until now, only Aldalur et al. [70] have reported an increase after pressing up to two months of ripening. Through HTS analysis, it has been observed that the starter *Lactococcus* remains dominant until the first month of ripening, but from that moment on other bacteria proliferate, mainly NSLAB [12]. Therefore, the lactic acid production by *Lactococcus* could lead to a decrease in the pH until 30 days of ripening, but the subsequent catabolism of lactate and amino acids by other microorganisms, such as NSLAB, would favour the aforementioned increase [18].

Compared to other raw ewe milk-derived cheeses, clear differences can be observed in gross composition [71,72]. Anyway, notable differences were observed even among the Idiazabal cheese samples elaborated by different producers, with pH and protein as the most differentiating parameters. This differentiation could be due to several reasons, such as differences in milk composition [73,74] or different cheese-making and ripening conditions [54,70]. For instance, Aldalur et al. [75] have reported differences in cooking pH and whey-draining pH between Idiazabal cheese producers. The type of rennet used or the concentration of coagulant enzyme employed have also been described as affecting the gross composition of cheese, including pH or protein content [76,77,78]. This could also explain the differences observed among producers, although it should be further studied in Idiazabal cheese.

Changes in the gross composition of cheese during ripening have been described as influencing the bacterial succession that takes place [18,79]. Based on the results obtained, gross composition evolution promoted LAB proliferation in Idiazabal cheese, especially that of *Lactobacillus* and to a lesser extent, that of *Streptococcus* and *Enterococcus*. However, the growth of environmental or non-desirable bacteria was negatively affected, mainly, that of *Psychrobacter* and to a lesser extent, that of *Erwinia*, *Pseudomonas* and *Pantoea*. In general, this would confirm that the evolution of the gross composition determines the bacterial dynamics during ripening, as previously proposed [12]. Specifically, the NaCl concentrations used are considered an important factor that controls the microbiological quality of cheese [18] and the results obtained could indicate that it is the most limiting factor for the proliferation of environmental and non-desirable bacteria in Idiazabal cheese. Within LAB, NaCl tolerance varies among genera and species. *Streptococcus* are able to grow until 2.5% of NaCl, whereas *Enterococcus* and some *Lactobacillus* species can grow up to 6.5% [18,80]. Consequently, the results obtained could indicate a great abundance of halotolerant *Lactobacillus* species and could explain why in a previous study for these same cheese samples the ripening time effect was only significant for this LAB [12]. On the other hand, *Streptococcus* and *Enterococcus* showed the highest positive correlations with the pH, confirming the previously observed succession between SLAB and NSLAB [12]. These correlations have also been described in other fermented products, although clear differences exist [81,82,83]. For the Suan zuo rou fermented meat, Wang et al. [82] have reported a positive correlation between *Lactobacillus* and pH and salt content, and a negative relation between pH and *Psychrobacter*, in line with the results of this study.

Regarding lipolysis, the concentration of most FFAs increased during ripening and saturated and short-chain FFAs predominated, which agrees with previous studies in Idiazabal cheese [19,69,84,85,86]. Individually, the predominance of C2, C4, C6 or C10 is also in accordance with what has been reported before [19,56,85,87]. Nevertheless, 4-methyl-C8, iC4 and iC6 were identified for the first time in Idiazabal cheese, as Amores et al. [88] have reported for iC5, C7, C11, C13 and C15. There are differences in the lipolysis process during ripening or in the predominant FFAs compared to other raw ewe milk-derived cheeses [89,90,91]. For instance, Esmaeilzadeh et al. [89] have reported a higher abundance of long-chain FFAs than short- or medium-chain FFAs during ripening of Kope cheese.

Clear differences were also observed in the lipolysis of Idiazabal cheese between producers, which could be due to several reasons. The use of artisanal rennet resulted in, mainly, higher concentrations of short- and medium-chain FFAs, with C4, C6, C8, C10 and C12 as characteristic. These observations agree with previous studies [19,69,85,86,92]. The lamb rennet employed for Idiazabal cheese production contains higher rennet pregastric lipase activity than commercial rennet [19]. This lipase has *sn*-3 stereospecificity and therefore, releases preferentially short-chain fatty acids since they are esterified on the *sn*-3 position of the TG of milk fat [17,88]. The lipase activity in artisanal rennets is variable among Idiazabal cheese producers [88], which could lead to differences in the concentrations of most individual FFAs in cheeses from different dairies. On the other hand, the LPL of raw milk is also an important lipolytic agent in raw milk cheeses, which releases preferentially short- and medium-chain fatty acids due to *sn*-1 and *sn*-3 stereospecificity [17]. However, LPL activity is very low in Idiazabal cheese [93]. For Friesian cross-bred sheep, it has been observed that an undernourishment or overfeeding of the herd affects the relative transcription accumulation of the genes involved in LPL biosynthesis [94].

Microbial lipases and esterases are considered important lipolytic agents in cheese [17,18]. The results obtained in the present study indicate that *Lactobacillus*, *Enterococcus* and *Streptococcus* are important lipolytic LAB in Idiazabal cheese. *Lactobacillus* was related to the release of medium- and long-chain FFAs, that is to say, it could present high lipase activity; whereas *Enterococcus* could present high esterase activity, as it was related to the release of short-chain FFAs [17,95]. Several lipases and esterases have been characterized from *Lactobacillus*, *Streptococcus* and *Enterococcus* species [96,97,98,99,100], which would support the results obtained. Nonetheless, no information has been found on the correlation between bacterial communities and the FFAs profile of fermented products, which has been more analysed with volatile or organic acids [40,41,46,101]. Furthermore, considering the importance that FFAs have in the sensory properties of Idiazabal cheese [19,85,86] and the differences observed in LAB composition among producers [12], these results could indicate an important role of LAB in sensory differentiation between producers that should be worth studying in depth.

Within environmental and non-desirable bacteria, the results obtained indicate an interesting lipolytic activity of *Obesumbacterium* that has not been described so far. This would make sense, since lipolytic activity has been previously described for other environmental or non-desirable bacteria, such as *Flavobacterium* or *Pseudomonas* [16,102,103,104]. Several negative correlations were observed between FFAs and environmental or non-desirable bacteria. FFAs can act as high-spectrum antimicrobial agents, comparable even with antimicrobial peptides [105,106,107]. Therefore, the results obtained could indicate that the release of FFAs in Idiazabal cheese could be part of the competitive inhibition mechanisms among bacteria. LAB could be the main bacterial genera responsible for the FFAs release and could have an inhibitory effect against environmental and non-desirable bacteria, mainly *Psychrobacter*, *Brevibacterium* and *Chromohalobacter*. This competitive inhibition mechanism could be one of the reasons why most environmental and non-desirable bacteria are inhibited during the first weeks of ripening [12]. Nonetheless, these correlations could also indicate the metabolic use of FFAs by *Psychrobacter*, *Brevibacterium* and *Chromohalobacter*, which has been little studied so far [108].

The increase in the total concentrations of BAs and the predominance of MMA, PUT, CAD and GABA during ripening partially agree with what has been reported for Idiazabal cheese [109] and other raw ewe milk-derived cheeses [110,111,112], since there are differences in the identified BAs and/or in their evolution during ripening. For instance, Ordóñez et al. [109] have identified isopenty1amine, spermidine, phenylethylamine and tryptamine in Idiazabal cheese and Tofalo et al. [110] have not identified HIS in Pecorino di Farindola cheese. The differences observed compared to previous studies and other raw ewe milk-derived cheeses, could be attributed, apart from the different BAs analysed, to the microbiota or to the different cheese-making or ripening parameters used by each producer [113,114]. Within the predominant BAs detected, information about MMA is scarce, but it has been described as harmful to livestock in high concentrations [115]. PUT and CAD, which are widely known toxic BAs, are in general below the limits considered toxic in cheese [26]. Finally, GABA predominance is of special interest due to its several beneficial effects, such as modulating sleep disorders, temporal and spatial memory [116], epilepsy [117], diabetes [118], depression [119] or cancer [120]. It is noteworthy that spermidine and spermine were not detected, which are endogenous BAs formed inherently by animals, plants or microorganisms and that are important for several physiological functions, such as neurotransmitter, vasoactive or regulating gene expression [24]. This could indicate that the identified BAs in Idiazabal cheese were mainly produced by bacterial decarboxylation [24]. It is well known that during cheese ripening there is an accumulation of FAAs as a result of secondary proteolysis [18], which has been observed for Idiazabal cheese [85,121] and other ewe milk-derived cheeses [110,122]. These FAAs serve as substrates for bacterial and/or endogenous decarboxylases, leading to an accumulation of BAs [123,124,125].

In terms of the functional relationship between bacterial communities and BAs production, *Lactobacillus* was strongly positively correlated with most of the identified BAs, while *Chromohalobacter* was positively correlated with MEA. SLAB, NSLAB and other microorganisms have been reported as decarboxylase-producing bacteria [28,126], which has been proven for several *Lactobacillus* strains, for example, *L. acidophilus* PNW3 [127,128,129,130], including halotolerant *Lactobacillus* species [131,132]. Body et al. [133] have reported the higher the salt concentration (up to 3%) the higher BAs production by *L. reuteri* strains, which is consistent with the results obtained in the present study. For other fermented foods, positive correlations between *Lactobacillus* and different BAs have also been reported, such as TYR, PUT or HIS [134,135], but results differ according to the product [136]. For *Chromohalobacter*, Jung et al. [137] have reported a positive correlation with PUT that would support the decarboxylase activity of this genus, but no information related to MEA has been found.

On the other hand, *Erwinia*, *Pantoea*, *Serratia*, *Ruminococcus*, *Bacillus*, *Raoultella* and also, *Chromohalobacter* and to a lesser extent *Lactobacillus*, showed some negative correlations with BAs, which could be related to BAs degradation abilities [138]. Although not for cheese, negative correlations have also been reported for other fermented products, for example, between *Erwinia* and HIS or *Pantoea* and TYR during spontaneous fermentation of pickled mustard tubers [134,135]. Nonetheless, BAs degradation ability has only been demonstrated for *Lactobacillus* and *Bacillus* species [139,140,141,142].

It is worth noting that the SLAB *Lactococcus*, which is the predominant genera during ripening [12], was not reported as key bacteria for any of the physico-chemical parameters studied. In other words, the evolution of the cheese quality and safety parameters studied would be related to the autochthonous microbiota of raw milk and not to the starter bacteria. Overall, these results could suppose interesting novel insights into dairy microbiology.

## 5. Conclusions

The present study investigated the dynamics of gross composition, FFAs and BAs and their relationship with bacterial communities during the ripening of raw ewe milk-derived Idiazabal cheese. All gross parameters (DM, protein, fat, Ca, Mg, P, and NaCl) except pH, FFAs and BAs showed an increasing trend during ripening. However, the producer also affected the gross composition and FFAs. In terms of functional relationships, *Lactobacillus*, *Streptococcus* and *Enterococcus* were positively correlated to gross composition and FFAs. This could indicate that their proliferation was favoured during ripening and that they were the main bacterial genera responsible for FFAs release, which could be important due to their aromatic impact. *Lactobacillus* was the unique LAB positively related to BAs production. Within environmental and non-desirable bacteria, 11 genera were reported as negatively correlated to gross composition, FFAs and BAs. This could be related, respectively, to the negative effect of gross composition evolution during ripening on their proliferation, to the antimicrobial effect of FFAs and/or the metabolic use of FFAs by these genera, and to BAs degradation capacities. Nonetheless, *Obesumbacterium* and *Chromohalobacter* were positively related to FFAs and BAs formation, respectively. Overall, this study presents novel knowledge to help understand the possible functional relationship between cheese microbiota and several physico-chemical parameters related to cheese quality and safety.

## Figures and Tables

**Table 1:** Mean concentration ± standard deviation (µmol/g) of total, unsaturated, saturated, short-, medium- and long-chain FFAs of Idiazabal cheese analysed from four producers (A, B, C and D) at six time points during ripening (1, 7, 14, 30, 60 and 120 days) (*n* = 48).

Producer	Ripening Time (Days)	Total FFAs	Unsaturated FFAs	Saturated FFAs	Short-Chain FFAs	Medium-Chain FFAs	Long-Chain FFAs
A	1	16.7 ± 4.36	0.331 ± 0.166	16.4 ± 4.19	15.4 ± 3.88	0.413 ± 0.0723	0.851 ± 0.412
	7	32.6 ± 2.26	0.481 ± 0.214	32.1 ± 2.05	30.6 ± 1.91	0.814 ± 0.00560	1.15 ± 0.342
	14	37.2 ± 4.92	0.486 ± 0.0122	36.7 ± 4.93	35.0 ± 4.64	1.04 ± 0.190	1.19 ± 0.0939
	30	41.2 ± 7.01	0.712 ± 0.0524	40.5 ± 6.96	38.1 ± 6.36	1.42 ± 0.429	1.66 ± 0.219
	60	56.4 ± 1.00	1.31 ± 0.497	55.1 ± 0.507	51.4 ± 0.463	2.10 ± 0.0919	2.81 ± 0.634
	120	77.8 ± 8.39	2.92 ± 0.434	74.9 ± 7.96	68.3 ± 6.96	3.50 ± 0.248	5.94 ± 1.18
B	1	6.04 ± 0.764	0.533 ± 0.0261	5.51 ± 0.738	4.47 ± 0.610	0.312 ± 0.0302	1.25 ± 0.124
	7	10.5 ± 2.51	0.584 ± 0.0288	9.93 ± 2.54	8.55 ± 2.01	0.465 ± 0.231	1.50 ± 0.267
	14	9.47 ± 0.508	0.474 ± 0.0623	8.99 ± 0.446	7.96 ± 0.563	0.321 ± 0.0689	1.19 ± 0.0141
	30	10.0 ± 0.431	0.655 ± 0.082	9.34 ± 0.513	8.21 ± 0.720	0.372 ± 0.0805	1.41 ± 0.209
	60	20.3 ± 10.6	0.831 ± 0.312	19.5 ± 10.3	17.9 ± 9.64	0.519 ± 0.207	1.90 ± 0.770
	120	18.1 ± 1.65	1.05 ± 0.0411	17.0 ± 1.61	15.3 ± 1.59	0.569 ± 0.0238	2.22 ± 0.0816
C	1	7.75 ± 1.30	0.227 ± 0.0409	7.52 ± 1.34	6.34 ± 1.65	0.476 ± 0.121	0.932 ± 0.23
	7	7.63 ± 1.96	0.273 ± 0.00353	7.36 ± 1.96	6.00 ± 1.70	0.568 ± 0.118	1.06 ± 0.145
	14	7.43 ± 1.61	0.240 ± 0.067	7.19 ± 1.55	5.92 ± 1.22	0.598 ± 0.148	0.913 ± 0.244
	30	16.3 ± 2.77	0.447 ± 0.0478	15.8 ± 2.72	14.0 ± 2.63	0.877 ± 0.00720	1.41 ± 0.142
	60	30.8 ± 4.16	0.512 ± 0.112	30.2 ± 4.05	27.8 ± 3.37	1.21 ± 0.366	1.70 ± 0.427
	120	52.1 ± 6.96	1.02 ± 0.0542	51.1 ± 7.01	47.2 ± 6.76	2.12 ± 0.123	2.83 ± 0.0777
D	1	17.5 ± 1.68	0.523 ± 0.132	17.0 ± 1.82	15.3 ± 1.72	0.719 ± 0.0749	1.51 ± 0.106
	7	21.2 ± 0.441	0.700 ± 0.107	20.5 ± 0.334	18.6 ± 0.173	0.816 ± 0.0865	1.72 ± 0.182
	14	22.6 ± 2.46	0.817 ± 0.188	21.8 ± 2.27	19.5 ± 2.23	1.09 ± 0.00966	2.03 ± 0.241
	30	36.1 ± 1.77	1.15 ± 0.427	35.0 ± 1.34	31.8 ± 1.26	1.60 ± 0.0392	2.74 ± 0.547
	60	38.4 ± 1.90	1.42 ± 0.179	37.0 ± 1.72	33.7 ± 1.53	1.73 ± 0.0656	3.02 ± 0.307
	120	51.6 ± 1.92	2.45 ± 0.0444	49.2 ± 1.87	43.4 ± 1.63	3.12 ± 0.145	5.04 ± 0.142
*p*-value ^1^	RT	***	***	***	**	**	***
	P	***	**	***	***	***	*

^1^ RT: ripening time factor effect; P: producer factor effect; * *p* ≤ 0.05, ** *p* ≤ 0.01, *** *p* ≤ 0.001.

## Data Availability

The data presented in this study are available on request from the corresponding author.

## Supplementary Materials

The following supporting information can be downloaded at: https://www.mdpi.com/article/10.3390/ani12223224/s1, Figure S1: Total concentrations of FFAs (µmol /g of cheese) accumulated during ripening time of Idiazabal cheese from four producers (A, B, C and D). Error bars represent the standard deviation of the measurements; Figure S2: CCorA analysis between key bacterial genera and gross composition parameters; Figure S3: CCorA analysis between key bacterial genera and FFAs; Figure S4: CCorA analysis between key bacterial genera and BAs; Table S1: Followed program for the separation of biogenic amines (BAs) from raw ewe milk-derived Idiazabal cheese samples (*n* = 48); Table S2: Mean concentration ± standard deviation of physico-chemical properties of the analysed Idiazabal cheeses from four producers (A, B, C and D) at six time points during ripening (1, 7, 14, 30, 60 and 120 days) (*n* = 48); Table S3: Mean concentration ± standard deviation (µmol/g) of individual FFAs identified in Idiazabal cheeses from four producers (A, B, C and D) at six time points during ripening (1, 7, 14, 30, 60 and 120 days) (*n* = 48); Table S4: Mean concentration ± standard deviation (µmol/g) of total and individual BAs identified in Idiazabal cheeses from four producers (A, B, C and D) at six time points during ripening (1, 7, 14, 30, 60 and 120 days) (*n* = 48).
