# Supplementary material for: Relationship between the Dynamics of Gross Composition, Free Fatty Acids and Biogenic Amines, and Microbial Shifts during the Ripening of Raw Ewe Milk-Derived Idiazabal Cheese
Source: Animals (Basel). 2022 Nov 21;12(22):3224. doi: 10.3390/ani12223224 (PMC9686631; doi:10.3390/ani12223224)
Supplement: Supplementary file 1 [file animals-12-03224-s001.zip › Figure S1.pdf]

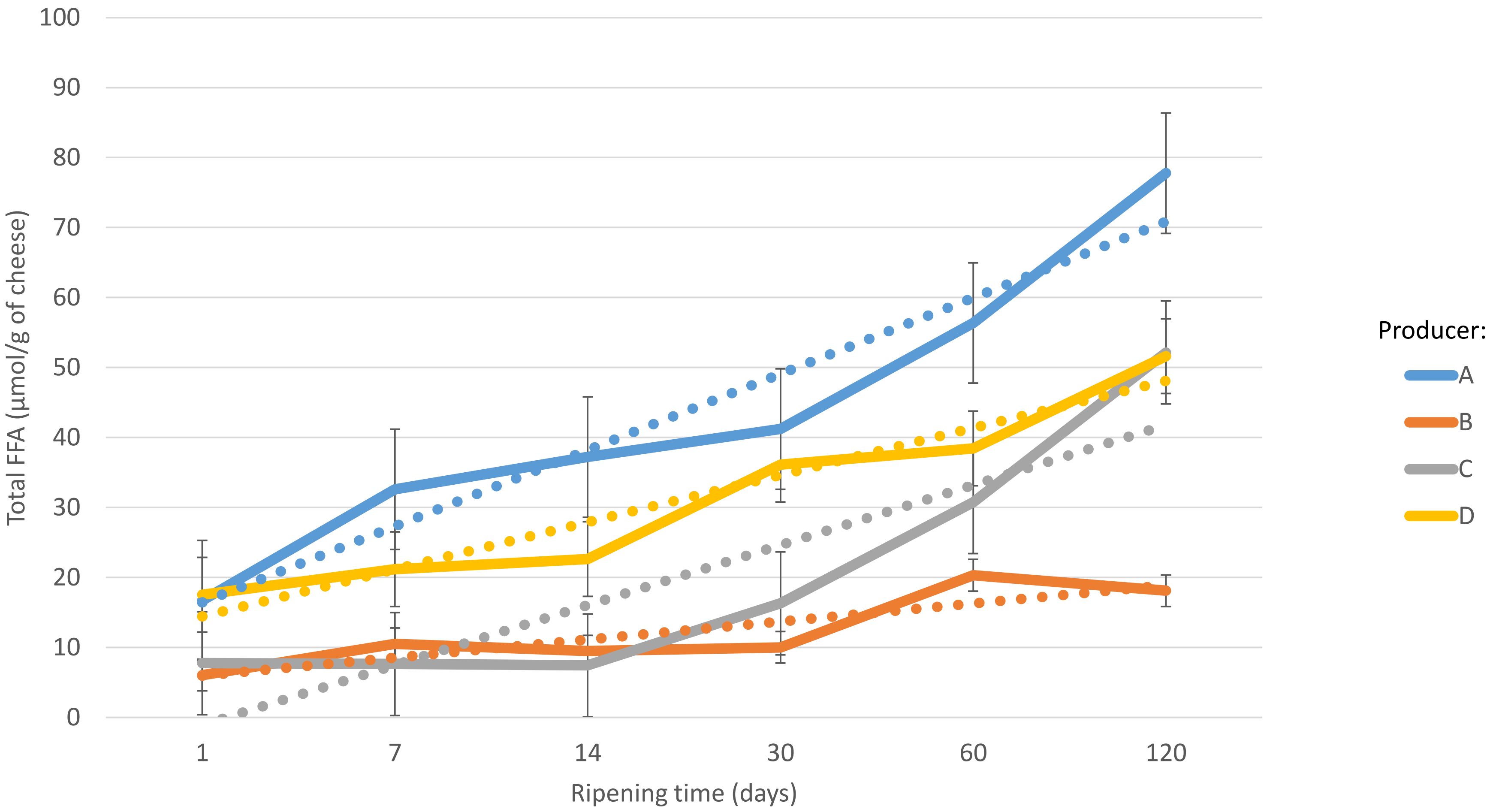

Figure S1. Total concentrations of FFAs (µmol/g of cheese) accumulated during ripening time of Idiazabal cheese from four producers (A, B, C and D). Error bars represent the standard deviation of the measurements.
