# Supplementary figures and images for: Relationship between the Dynamics of Gross Composition, Free Fatty Acids and Biogenic Amines, and Microbial Shifts during the Ripening of Raw Ewe Milk-Derived Idiazabal Cheese
Source: Animals (Basel). 2022 Nov 21;12(22):3224. doi: 10.3390/ani12223224 (PMC9686631; doi:10.3390/ani12223224)

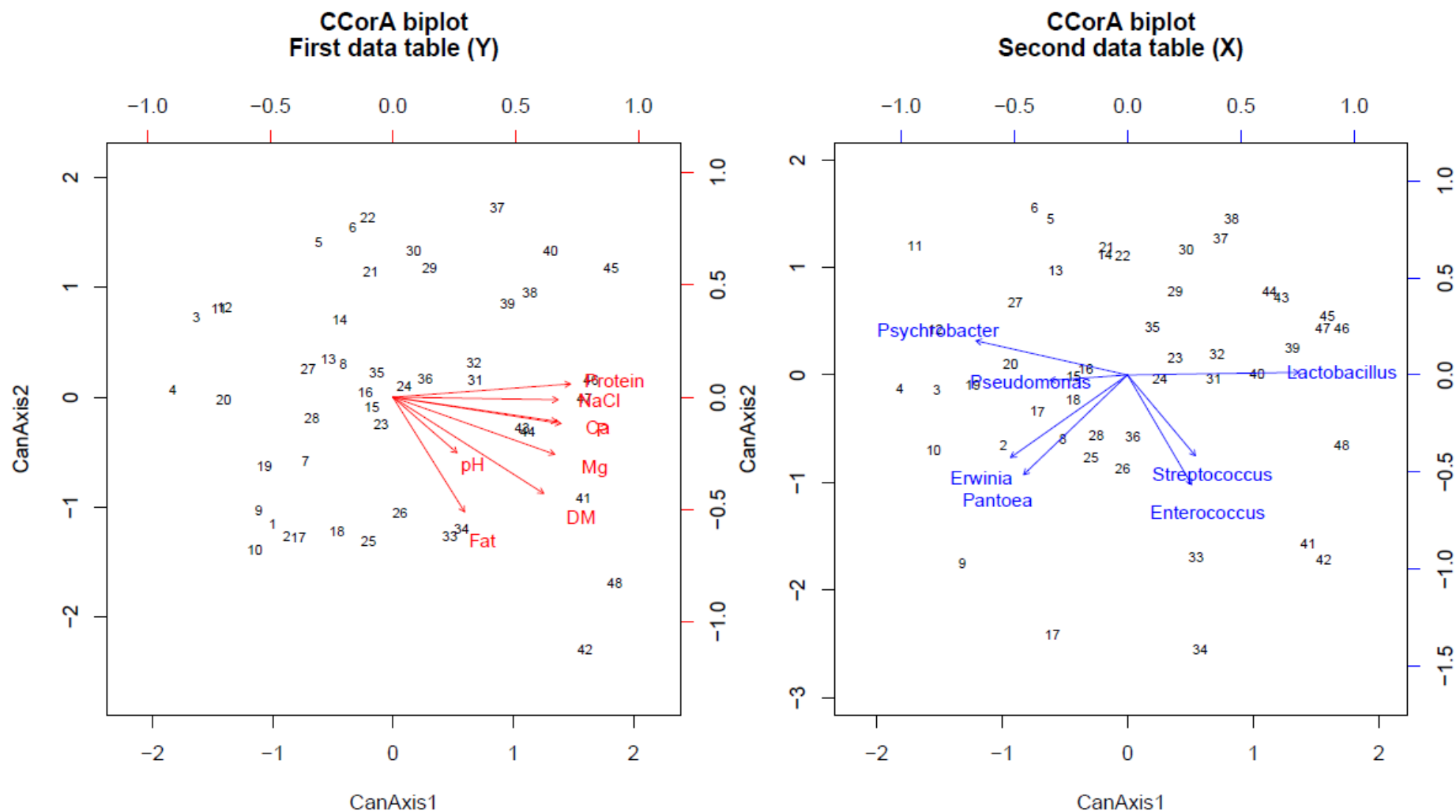

**Figure S2.** CCorA analysis between key bacterial genera and gross composition parameters.

Supplement: Supplementary file 1 [file animals-12-03224-s001.zip › Figure S2.pdf]

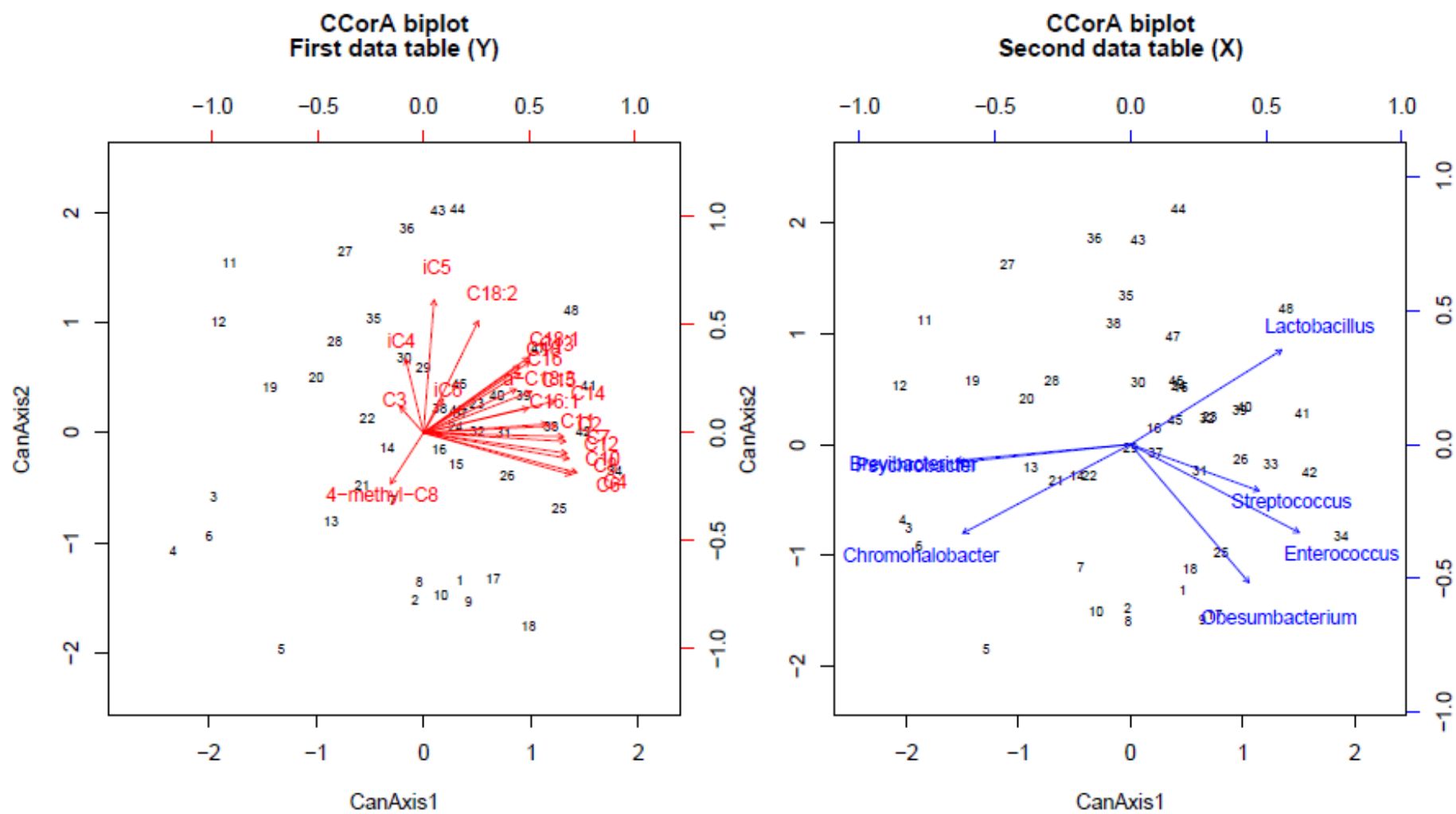

**Figure S3.** CCorA analysis between key bacterial genera and FFAs.

Supplement: Supplementary file 1 [file animals-12-03224-s001.zip › Figure S3.pdf]
